# MR Imaging of SCA3/MJD

**DOI:** 10.3389/fnins.2020.00749

**Published:** 2020-08-04

**Authors:** Na Wan, Zhao Chen, Linlin Wan, Beisha Tang, Hong Jiang

**Affiliations:** ^1^Department of Neurology, Xiangya Hospital, Central South University, Changsha, China; ^2^National Clinical Research Center for Geriatric Disorders, Xiangya Hospital, Central South University, Changsha, China; ^3^Key Laboratory of Hunan Province in Neurodegenerative Disorders, Central South University, Changsha, China

**Keywords:** SCA3/MJD, MR imaging, morphometric MRI, diffusion tensor imaging, blood oxygen level-dependent functional MRI, magnetic resonance spectroscopy

## Abstract

Spinocerebellar ataxia type 3/Machado–Joseph disease (SCA3/MJD) is a progressive autosomal dominantly inherited cerebellar ataxia characterized by the aggregation of polyglutamine-expanded protein within neuronal nuclei in the brain, which can lead to brain damage that precedes the onset of clinical manifestations. Magnetic resonance imaging (MRI) techniques such as morphometric MRI, diffusion tensor imaging (DTI), functional magnetic resonance imaging (fMRI), and magnetic resonance spectroscopy (MRS) have gained increasing attention as non-invasive and quantitative methods for the assessment of structural and functional alterations in clinical SCA3/MJD patients as well as preclinical carriers. Morphometric MRI has demonstrated typical patterns of atrophy or volume loss in the cerebellum and brainstem with extensive lesions in some supratentorial areas. DTI has detected widespread microstructural alterations in brain white matter, which indicate disrupted brain anatomical connectivity. Task-related fMRI has presented unusual brain activation patterns within the cerebellum and some extracerebellar tissue, reflecting the decreased functional connectivity of these brain regions in SCA3/MJD subjects. MRS has revealed abnormal neurochemical profiles, such as the levels or ratios of N-acetyl aspartate, choline, and creatine, in both clinical cases and preclinical cases before the alterations in brain anatomical structure. Moreover, a number of studies have reported correlations of MR imaging alterations with clinical and genetic features. The utility of these MR imaging techniques can help to identify preclinical SCA3/MJD carriers, monitor disease progression, evaluate response to therapeutic interventions, and illustrate the pathophysiological mechanisms underlying the occurrence, development, and prognosis of SCA3/MJD.

## Introduction

**FIGURE 1:**
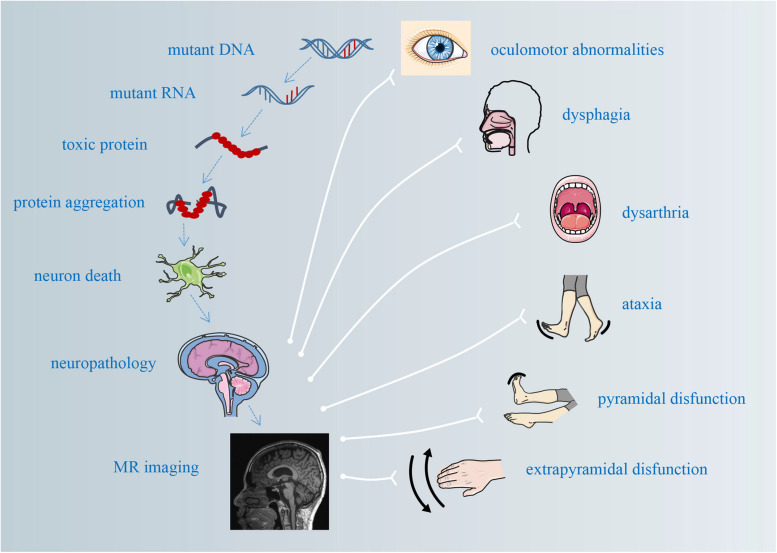
The pathological mechanisms underlying atrophy and its correlations with clinical manifestations.

Spinocerebellar ataxias (SCAs) are a group of autosomal dominantly inherited progressive neurodegenerative disorders with obvious clinical and genetic heterogeneity (Klockgether et al., 2019). To date, more than 40 genetically distinct SCA subtypes have been identified (Klockgether et al., 2019). Spinocerebellar ataxia type 3, also known as Machado–Joseph disease (SCA3/MJD), is the most common type of autosomal dominantly inherited cerebellar ataxia (Tang et al., 2000; Jiang et al., 2005; Costa Mdo and Paulson, 2012; Chen et al., 2018b). The characterized manifestations include not only progressive cerebellar ataxia but also pyramidal and extrapyramidal signs, such as dysarthria, dysphagia, dystonia, peripheral neuropathy, and oculomotor abnormalities (Schöls et al., 2004; Costa Mdo and Paulson, 2012). SCA3/MJD is caused by unstable cytosine–adenine–guanine (CAG) repeats located in the 10th exon of the ATXN3 gene (Kawaguchi et al., 1994; Schöls et al., 2004; Chen et al., 2018a). The number of repeats in the mutant ATXN3 allele ranges from 52 to 91 in SCA3/MJD patients, which results in the expansion of a polyglutamine tract within the ATXN3 protein (Todd and Paulson, 2010; Souza et al., 2016). The accumulation and the aggregation of expanded polyglutamine stretches within the nuclei of neurons in susceptible brain regions might induce direct or indirect neurotoxic effects that eventually lead to neuron loss and brain atrophy (Yamada et al., 2001; Costa Mdo and Paulson, 2012). These abnormalities in neuronal nuclei involve both cortical and subcortical regions, including the cerebral cortex, basal ganglia, thalamus, cerebellum, and brain stem, as observed in previous neuropathological and histopathological studies (Durr et al., 1996; Yamada et al., 2001; Schöls et al., 2004). These brain lesions may be responsible for the motor and the mental dysfunctions identified in SCA3/MJD patients in previous clinical and neuroradiological studies (Klockgether et al., 2019; Figure 1). The earliest pathophysiological changes precede the appearance of ataxia symptoms in preclinical SCA carriers (Maas et al., 2015), and detailed knowledge of the preclinical stage is of great significance for a more comprehensive understanding of the pathogenesis of SCAs. Two primary scales currently used to quantify the motor defects of ataxia in natural history studies of SCAs are the Scale for the Assessment and Rating of Ataxia (SARA) (Schmitz-Hubsch et al., 2006) and the International Cooperative Ataxia Rating Scale (ICARS) (Trouillas et al., 1997), both of which are semi-quantitative and have been extensively validated as useful tools for ataxia severity assessment (D’Abreu et al., 2007); the higher the total score, the worse is the patient’s ataxic syndrome. In addition, the total score has been shown to be correlated with measures of the quality of life in patients with SCAs (Saute et al., 2012). However, these scales lack sensitivity in the early stages of SCA, including in the preclinical stages when disease-modifying therapies and neuroprotective agents are likely to be most effective (Rubinsztein and Orr, 2016), and usually have poor test–retest reliability (Mueller et al., 2006). Consequently, non-invasive and objective biomarkers should supplement clinical scales to directly identify subtle brain abnormalities in individuals before the onset of ataxia and assess the treatment effects of therapeutic interventions. Neuroimaging techniques have shown promising results in the investigation of brain damage with high accuracy and reproducibility. Since SCAs have extremely low prevalence rates (Schulz et al., 2010) and histopathological data are scarcely available for most degenerative ataxias, magnetic resonance (MR)-based structural and functional imaging techniques have gained growing attention in the exploration of pathogenic mechanisms and potential biomarkers that can indicate disease progression even in the preclinical stage of neurodegenerative diseases such as SCA (Dohlinger et al., 2008; D’Abreu et al., 2010; Baldarcara et al., 2015; Mascalchi and Vella, 2018). Structural or functional abnormalities of the brain tissue can be observed by different MR imaging techniques, including morphometric magnetic resonance imaging (MRI), diffusion tensor imaging (DTI), blood oxygen level-dependent functional MRI (BOLD fMRI), and magnetic resonance spectroscopy (MRS). The utility of these techniques constitutes a potential source of neuroimaging biomarkers, which can be used to identify potential pathogenesis underlying both manifested and preclinical SCA patients, assess disease severity and progression, monitor therapeutic effects, and determine nuances that can be used as endpoints for future clinical trials. Given this scenario, the purpose of this review was to summarize the main MR imaging properties and their correlation with clinical features in manifested and preclinical SCA3/MJD patients, which could help to assess the value of MR imaging indicators as diagnostic, disease progression, and surrogate biomarkers for SCA3/MJD.

## Morphometric MRI

Morphometric MRI has become a standard method to monitor brain morphological alterations in neurodegenerative diseases. In patients with SCAs, the main focus has been the visualization and the quantification of brain volume changes that particularly affect the cerebellum and the brainstem. Patterns of atrophy in different SCA genotypes are specific but sometimes overlapping. Voxel-based morphometry (VBM) allows a quantitative assessment and automatic unbiased measurement of brain tissue density and volume with high accuracy and repeatability.

### Widespread Alterations in Brain Morphology in SCA3/MJD Patients

**FIGURE 2:**
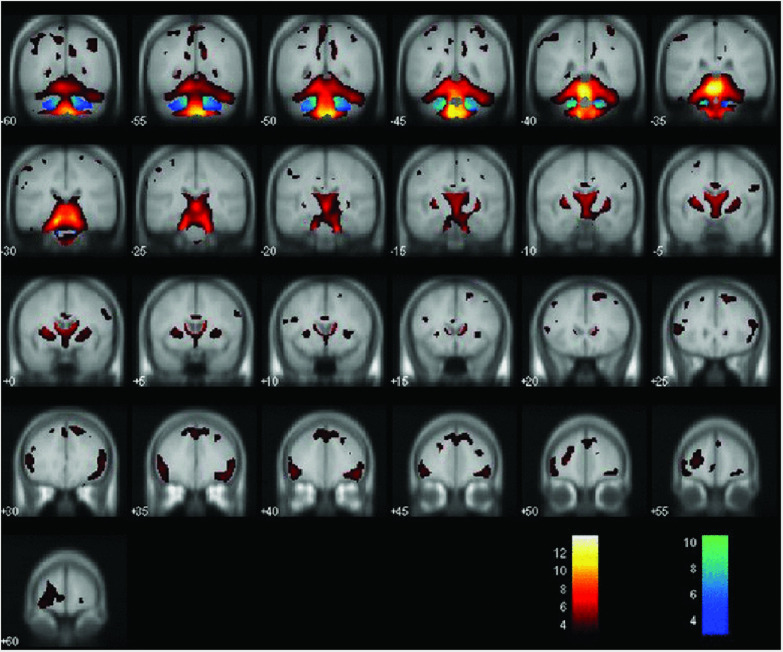
Areas of decreased gray matter density (hot colors) and white matter density (cold colors) when comparing MJD/SCA3 and controls (*P* < 0.001). Reprinted with permission from Wiley (D’Abreu et al., 2012).

Previous conventional MRI study using manual segmentation method has revealed widespread lesions in the brain structure of SCA3/MJD patients, including those in the cerebellum, brain stem, cerebral cortex, and basal ganglia (Klockgether et al., 1998). Morphometric MRI studies using VBM analysis allow a quantitative and automatic assessment of brain atrophy in the cerebellum, brainstem, basal ganglia, thalamus, and distinct cortical areas, as shown in Figure 2 and summarized in Table 1. Widespread reductions in cortical gray matter density validated the atrophy of the whole brain (D’Abreu et al., 2012). To quantify the shape complexity of cortical folding, three-dimensional fractal dimension method was applied in the assessment of cortical degeneration, and reduced cerebral complexity more extensive than traditional olivopontocerebellar regions and the corticocerebellar system was detected (Wang T. Y. et al., 2015; Wang et al., 2020; Huang et al., 2017). Besides that, the involved cortex widely overlaps with cerebellum-related cortex and basal ganglia-related cortex (Wang et al., 2020). Using volumetric measurements, SCA3/MJD patients showed extensive atrophy of the cerebral cortex, thalamus, and basal ganglia, as well as the cerebellum and the brainstem (Rezende et al., 2018). The cerebellum and the brainstem, compared with the other abovementioned regions, appear to be more severely affected in SCA3/MJD patients (Lukas et al., 2006; Schulz et al., 2010; Eichler et al., 2011; Kang et al., 2014; Hernandez-Castillo et al., 2017; Rezende et al., 2018; Peng et al., 2019). Susceptibility-weighted imaging revealed atrophy of the cerebellar nuclei in SCA3/MJD patients (Stefanescu et al., 2015), and a hyperintense signal of the transverse pontine fibers was observed by T2-weighted and/or proton-weighted axial MRI in 14 out of 31 SCA3 patients (Murata et al., 1998) as well as in one other postmortem-confirmed case (Imon et al., 1998), which are all consistent with an olivopontocerebellar atrophy pattern as demonstrated in neuropathologic studies (Durr et al., 1996; Schöls et al., 2004).

**TABLE 1:** Summary of baseline data of the affected brain regions detected with voxel-based morphometry analysis.

References	Sample size	Age	AOO	DD	CAG	Ataxia scale	Ataxia score	MRI scanner	Magnetic flux density	Head coil	Cere- bellum	Brain stem	Cere- brum	Basal ganglia	Thalami	Limbic system
Peng et al. (2019)	31	38.91	34.88	4.81	71.84	International Cooperative Ataxia Rating Scale (ICARS)	26.81	Sonata, Siemens	1.5 T	–	√	√	√			√
Meles et al. (2018)	17	45.3	35.6	9.7	70	Scale for the Assessment and Rating of Ataxia (SARA)	10	Achieva, Philips	3 T	32-channel	√		√	√		
Hernandez-Castillo et al. (2017)	17	40.41	33.47	6.94	-	SARA	13.26	Achieva, Philips	3 T	32-channel	√	√	√			
Duarte et al. (2016)	13	43.66	32.94	7.51	65.67	SARA	11.92	Magnetom TIM Trio, Siemens	3 T	12-channel	√	√	√		√	√
Kang et al. (2014)	12	50.5	–	11.0	70.6	SARA	10.3	Magnetom TIM Trio, Siemens	3 T	8-channel	√	√			√	
Guimarães et al. (2013)	38	40.38	40.02	9.3	68.08	ICARS/SARA	32.08/14.65	Achieva, Philips	3 T	–	√	√				
Lopes et al. (2013)	32	46.78	36.72	10.09	69.0	SARA	13.6	Achieva, Philips	3 T	–	√		√	√		√
D’Abreu et al. (2012)	45	47.02	37.04	9.97	72	ICARS	36.36	Elscint Prestige	2 T	–	√	√	√	√	√	√
Goel et al. (2011)	10	38.0	-	5.8	–	ICARS	48.6	Magnetom Vision Plus, Siemens	1.5 T	–	√		√			√
Schulz et al. (2010)	24	47.3	-	11.7	–	SARA	12.0	Sonata, Siemens	1.5 T	–	√	√				
Nanri et al. (2010)	4	73	-	12	–	–	–	Magnetom Symphony, Siemens	1.5 T	–	√	√				
Lukas et al. (2006)	9	53	47	–	64	ICARS	30	Magnetom Symphony, Siemens	1.5 T	–	√	√	√			

*AOO, age of onset; DD, disease duration.*

Volume reductions in the basal ganglia area of SCA3/MJD patients have also been reported (Klockgether et al., 1998; Murata et al., 1998; de Rezende et al., 2015; Rezende et al., 2018). Pallidal atrophy was found in two patients whose disease durations were over 10 years (Tokumaru et al., 2003), and significant decreases in gray matter volume were observed in the putamen and the pallidum (Lopes et al., 2013; Reetz et al., 2013). Some studies have failed to observe atrophy of the basal ganglia, which might be attributable to the relatively short disease duration of the recruited patients (Peng et al., 2019).

In order to find out whether cerebral cortex is affected in SCA3/MJD patients and to find the clinical and neuropathological connections with these regions, de Rezende et al. (2015) collected MRI data of 3D-T1 MR images of 49 patients with SCA3/MJD. The cortical thickness and the volume of subcortical nuclei were calculated by FreeSurfer, widespread cortical and subcortical atrophy was found (de Rezende et al., 2015). VBM studies also confirmed the extensive reduction in the volume of gray matter in the brain cerebral cortex, including the frontal, parietal, temporal, and occipital lobes (Goel et al., 2011; D’Abreu et al., 2012; Duarte et al., 2016). In addition, significant loss of both gray and white matter in the thalamus has been reported by VBM (Kang et al., 2014; Duarte et al., 2016), which is consistent with the decreased thalamic volumes in SCA3/MJD patients when compared to controls using both automated measurements and manual segmentation methods (D’Abreu et al., 2011).

A longitudinal study with a large number of subjects showed that the gray matter density of multiple brain regions other than the cerebellum was also a strong determinant of the final ICARS score (D’Abreu et al., 2012), which was a strong supporting information for the widespread brain pathology in SCA3/MJD. A correlation of the atrophy profiles in the cerebellum and the brain stem with the SARA/ICARS total scores and disease duration of SCA3/MJD patients has been found (Liang et al., 2009; Hernandez-Castillo et al., 2017; Peng et al., 2019), suggesting that these regions account for the main clinical features found in SCA3/MJD patients. In addition, a previous study using multimodal MR imaging-based analyses in a large cohort of patients and presymptomatic subjects showed that volume measurements were more sensitive than SARA (Reetz et al., 2013), which provides a strong empirical basis for using these objective neuroimaging biomarkers to supplement ataxia assessment scales in the evaluation of clinical outcomes. Some neuroimaging studies have demonstrated an inverse correlation between the CAG repeats length and the volume of brainstem, cerebellum, and basal ganglia (Yoshizawa et al., 2003; Schulz et al., 2010; Camargos et al., 2011; Peng et al., 2019), whereas some failed to reveal a significant association between the CAG repeat expansion and the volume changes in the specific brain region (Burk et al., 1996; Klockgether et al., 1998; Eichler et al., 2011; Goel et al., 2011; Guimarães et al., 2013; Stefanescu et al., 2015; Huang et al., 2017). Apart from these neuroimaging reports, a pathoanatomic study also disclosed no correlation between the degeneration of the cerebellar Purkinje cell layer or the deep cerebellar nuclei with the CAG repeat length (Scherzed et al., 2012). Considering that several studies have demonstrated the inverse correlation between CAG repeat length and age of onset as well as disease severity, the rapid cerebellar degeneration in SCA3 patients with earlier disease onset may not be caused by the longer CAG repeat length alone (Huang et al., 2017).

### Brain Morphology Abnormalities Progress From Infratentorial to Supratentorial Areas

Brain morphology abnormalities are initially observed in the infratentorial areas early in the disease course. Studies in preclinical SCA3 patients have detected volumetric reductions in the brainstem, substantia nigra, and spinal cord (Rezende et al., 2018). Jacobi et al. (2013) also recorded a mild loss of brainstem volume in a cohort of nine preclinical European SCA3/MJD carriers, although the result was non-significant after a statistical analysis, which was in line with the results of a recent MRS study that demonstrated reduced N-acetyl aspartate (NAA)/myo-inositol (mI) ratios in the pons of preclinical SCA3/MJD carriers (Joers et al., 2018). These observations indicated that brain structural and functional damage begins in the early stages and precedes the onset of clinical manifestations of SCA3/MJD, which is commonly observed in neurodegenerative disorders, such as Huntington’s disease, Alzheimer’s disease, and familial amyotrophic lateral sclerosis (Ramani et al., 2006; Faria et al., 2016; Lee et al., 2017).

Later in the disease course, structural damage diffusely proceeds to the supratentorial areas. Mild frontal atrophy and pallidal atrophy were observed in two patients whose disease durations were over 10 years (Tokumaru et al., 2003). High-intensity change along the internal capsules was also detected by T2-weighted MR image in an autopsied case who died at aged 60, which was 19 years after her initial symptoms, and the autopsy findings suggested neuronal loss, astrocytosis, and gliosis in the internal segment of the globus pallidus (Horimoto et al., 2011). Wang T. Y. et al. (2015) detected abnormal electroencephalogram signals in the cerebral cortex of SCA3 patients in the late stage of the disease. In another study, extensive atrophy of the basal ganglia and cerebral cortex was also observed in the late stages of SCA3/MJD (Rezende et al., 2018), which was consistent with the dementia and delirium symptoms in four SCA3/MJD patients in the late stages (Ishikawa et al., 2002).

So far, the most commonly used clinical outcome and disease severity assessment measures in natural history studies and clinical trials of SCAs are ataxia rating scales such as SARA and ICARS, However, both of them are semiquantitative and lack sensitivity in the earliest stages of SCAs, including the preclinical stages when brain structural damage has already taken place (Rezende et al., 2018). Although such scales are essential for disease progression assessment, they should be supplemented with quantitative and non-invasive neuroimaging data to directly assess the neuroanatomic alteration of the brain. Meanwhile, the use of MR imaging technique in the late stages of SCA3/MJD may be limited, as shown in the longitudinal study that no progression of atrophy was detected after an interval of approximately 1 year, which might be caused by the floor effect or short duration of follow-up (D’Abreu et al., 2012). Thus, a combination of both clinical scales and MR imaging techniques or even body fluid biomarkers will allow a better reflection of the disease trajectory and a larger effect size than any biomarker alone, which will enable an accurate evaluation of disease progression and brain atrophy staging in neurodegenerative diseases, especially in rare diseases like SCA where patient recruitment is challenging. Besides that, as discovered by Rezende et al. (2018) a typical caudal–rostral progression of brain structural damage in SCA3/MJD patients suggested a potential staging scheme for SCA3/MJD like the Braak staging scheme of brain pathology for Parkinson’s disease. Further longitudinal studies with postmortem histology data are needed to validate this hypothesis.

### The Correlation of Brain Atrophy With Motor and Cognitive Impairments in SCA3/MJD Patients

The cerebellum plays an important role not only in motor control but also in non-motor functions such as language, memory, and visual ability (Wolf et al., 2009). Stoodley and Schmahmann (2010) illustrated that when the anterior lobe of the cerebellum and parts of the cerebellum lobule VI are damaged, communication between the cerebellar motor system and the cerebral and spinal motor systems is interrupted, which can result in cerebellar motor syndrome. In addition to its role in motor coordination, the cerebellum is also involved in cognitive processing and emotional control (Schmahmann and Caplan, 2006). When lesions involve the posterior lobe of the cerebellum and vermis, the cerebral cognitive system modulated by the cerebellum may be disrupted, which can lead to cerebellar cognitive affective syndrome (CCAS) (Schmahmann and Caplan, 2006; Stoodley and Schmahmann, 2010). Many studies have demonstrated the occurrence of cognitive deficits, including executive impairments, such as verbal fluency and verbal memory deficits, and impairments in spatial cognition, including visuospatial function, visual memory, visuoconstruction, and visual attention, and emotional deficits in SCA3/MJD patients (Maruff et al., 1996; Zawacki et al., 2002; Kawai et al., 2004; Klinke et al., 2010; Lopes et al., 2013; Roeske et al., 2013; Braga-Neto et al., 2014), which are in accordance with the characteristic manifestations of CCAS (Schmahmann and Sherman, 1998; Schmahmann, 2004; Schmahmann and Caplan, 2006; Braga-Neto et al., 2014). Widespread cerebral cortical atrophy was also correlated with motor (de Rezende et al., 2015) and cognitive (Lopes et al., 2013) impairments. Occipital damage is specifically associated with visuospatial deficits, and the cingulate gyri plays an important role in motor control and the regulation of cognition (Paus, 2001; de Rezende et al., 2015). Atrophy of the hippocampus may partly explain the visual and verbal memory impairments observed in some SCA3/MJD patients (de Rezende et al., 2015).

The basal ganglia are a group of subcortical nuclei that interconnect with the thalamus and the cerebral cortex by several parallel reentrant loops and are primarily responsible for motor control (Alexander et al., 1986; Alexander and Crutcher, 1990). Information flows back to the cortex through two functional opposing pathways from the basal ganglia to precisely perform the movement (Lanciego et al., 2012). On the one hand, the motor circuit originates from several frontal lobe regions and mainly projects to the putamen. After intermediate processing, the information is returned to the neocortex through the ventrolateral part of the thalamus (Riecker et al., 2003). On the other hand, the striatum can also target the output stage of the basal ganglia through the external segment of the globus pallidus and the subthalamus (Riecker et al., 2003; Lanciego et al., 2012). Basal ganglia abnormalities can lead to dystonia and parkinsonism, however, parkinsonian motor features seem not to be that common in SCA3/MJD patients since the degeneration of the motor territory of the subthalamic nucleus can partially ameliorate the manifestation of parkinsonism in SCA3/MJD patients (Schols et al., 2015). The correlation between brain atrophy patterns and ataxia has also been found in SCA1, SCA6 (Schulz et al., 2010), SCA7 (Hernandez-Castillo et al., 2016), and SCA17 (Reetz et al., 2010) patients, suggesting that these clinical manifestations may be caused by the same atrophy pattern.

However, most of the previous SCA3/MJD brain morphological studies are macroscopic changes of gray and white matter (such as the volume of gray and white matter, etc.), and the changes of brain microstructure are not very clear. In recent years, the rapid development of neuroimaging technology, especially DTI technology, provides the possibility for the study of the microstructural integrity of white matter *in vivo*.

## Diffusion Tensor Imaging

Water diffuses mainly along the major axis of the white matter (WM) fiber tract in brain tissue (Moseley et al., 1990). Therefore, by measuring the directionality of water diffusion (fractional anisotropy, FA) (Basser and Pierpaoli, 1996) and three other main parameters, including axial diffusivity (AD), radial diffusivity (RD), and mean diffusivity (MD), DTI has become a widely used quantitative and non-invasive methodology to assess the microstructure and the integrity of the brain WM fiber tracts (Le Bihan, 2003). Tract-based spatial statistics (TBSS), using non-linear image transformation, is a widely applied technique in DTI analysis that can combine the strength of both voxel wise and tractography-based analyses (Smith et al., 2006). By measuring the anisotropic diffusion of water in the WM tract, TBSS can provide information about anatomical connectivity in the brain (Yeh et al., 2009).

### Decreased FA and Increased AD, RD, and MD in SCA3/MJD Patients

**FIGURE 3:**
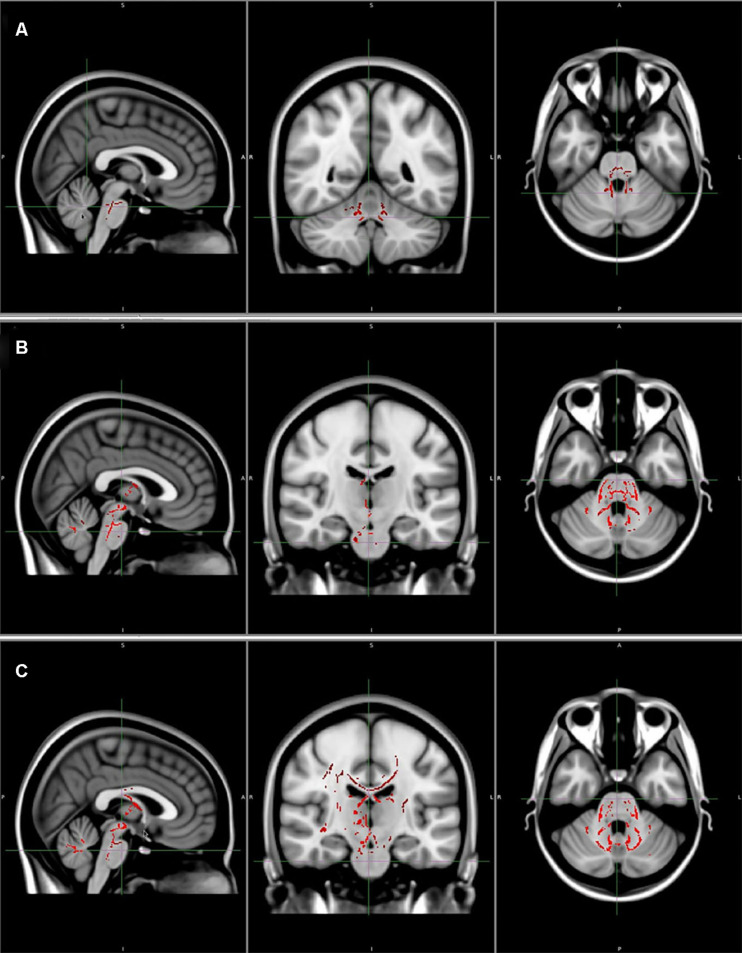
**(A)** Areas of reduced fractional anisotropy (*P* < 0.05, corrected). **(B)** Areas of increased axial diffusivity (*P* < 0.05, corrected). **(C)** Areas of increased radial diffusivity (*P* < 0.05, corrected). Reprinted with permission from Wiley (Guimarães et al., 2013).

DTI studies with TBSS have revealed a widespread decrease in FA accompanied by increased AD, RD, and MD in the WM across the whole brain, especially the cerebellum and the brainstem, as well as some supratentorial areas, such as the bilateral frontal, parietal, temporal, and occipital lobes and thalamus, in patients with SCA3/MJD compared with those in controls, as shown in Figure 3 (Guimarães et al., 2013; Nunes et al., 2015; Jao et al., 2019). What is more, MD increases showed a similar pattern as FA decreases (Kang et al., 2014; Wu et al., 2017; Jao et al., 2019), and these findings were in line with the results from the multi-atlas segmented findings (Rezende et al., 2018). WM abnormalities were also identified in some fiber pathways that were mostly in the cerebellar connecting tracts, including the pyramidal tract, thalamic radiations, medial lemnisci, corticospinal tract, corticobulbar tract, and corticopontocerebellar tract in patients with SCA3/MJD (Rezende et al., 2018; Jao et al., 2019). WM tract impairments are common features among SCA patients. Decreased FA and increased MD in the cerebellar peduncles, the bilateral posterior limb of the internal capsule, and the corona radiata have been observed in SCA2 patients (Hernandez-Castillo et al., 2015), and widespread FA reductions beyond the cerebellum and the pons have also been found in SCA7 patients (Alcauter et al., 2011).

### WM Tracts Are Partially Impaired in the Preclinical Stage of SCA3/MJD

SCA3/MJD is a progressive neurodegeneration disease with brain pathology preceding the onset of clinical symptoms. The discovery of the microstructural changes of preclinical SCA3/MJD is helpful to find neuroimaging markers and even predict the clinical onset of disease. Wu et al. (2017) identified decreased FA values and increased MD in the cerebellar peduncles of preclinical SCA3/MJD patients. A multi-atlas analysis in another study revealed that all DTI-based parameters were abnormal in the cerebellar peduncles of asymptomatic SCA3/MJD gene carriers (Rezende et al., 2018). WM impairments in the cerebellum were also discovered in preclinical patients with SCA1 (Yoo and Oh, 2017) and SCA6 (Falcon et al., 2016). The cerebellar peduncles are responsible for both efferent and afferent information. The inferior cerebellar peduncles consist of olivocerebellar and dorsal spinocerebellar tracts and are mainly involved in the coordination of movement and proprioception, receiving movement information and sensory information related to movement (Tokumaru et al., 2003). The middle cerebellar peduncles mainly receive afferent fibers from the pontine nuclei to the cerebellum, which make up most of the corticopontocerebellar tract, bringing information from the cerebral cortex (Keser et al., 2015). The superior cerebellar peduncles contain mostly efferent fibers that originate from the cerebellar nuclei for the connections of the cerebellum and the mesencephalon and the cerebrum (Johna, 2006; Dayan et al., 2016). These abnormalities in the cerebellar peduncles in preclinical SCA patients exemplify that the afferent and efferent systems of the cerebellum have already been damaged in the early disease course of SCA.

### WM Lesions Are Related to Disease Severity in SCA3/MJD Patients

Abnormal TBSS results were inversely correlated with SARA scores, ICARS scores, and neuropsychological assessment results of SCA3/MJD patients, which indicated that damage to the WM tracts across specific regions may be closely related to disease severity in SCA3/MJD patients, including the severity of movement disorders (Kang et al., 2014; Peng et al., 2019) and cognitive dysfunction (Lopes et al., 2013; Wu et al., 2017). WM plays a vital role in connecting various brain regions and coordinating the communication between them, while no significant correlation was found between any of the DTI parameters and the number of CAG repeats in expanded alleles (Guimarães et al., 2013; Kang et al., 2014; Peng et al., 2019).

DTI-derived AD and RD have been demonstrated to be promising biomarkers of axonal injury and myelin abnormalities, respectively, in mouse models of white matter injuries (Song et al., 2002, 2003, 2005). Consistent with the DTI changes in SCA3/MJD, DTI-derived AD and RD increased were also detected in different types of multiple sclerosis lesions (Naismith et al., 2013; Wang Y. et al., 2015). It has been suspected that the changes of AD may reflect the changes of axons in a time-dependent way, characterized by initial decrease when axonal degradation is expected during the acute phase and then normalization or even increase with the removal of axonal fragments (Concha et al., 2006), while the extent of increased RD reflects the severity of demyelination and the increased freedom of cross-fiber diffusion in white matter (Song et al., 2002, 2005). Thus, the increase of AD and RD found in SCA3/MJD patients supports the hypothesis of the coexistence of chronic axonal damage and extensive myelin degeneration as observed in experimental models of multiple sclerosis and other white matter injuries. As previously mentioned, FA indicates the directionality of water diffusion (Pierpaoli and Basser, 1996), and reduced FA can be caused by the degradation of axonal membranes, i.e., myelin sheaths (Werring et al., 2000; Pierpaoli et al., 2001), and reductions in axonal fiber density (Takahashi et al., 2002; Beaulieu, 2009). MD is the overall mean-squared displacement of water molecules restricted by organelles and membranes (Neil et al., 1998), which reflects the diffusivity of water based on cellular density and extracellular volume (Gass et al., 2001; Le Bihan et al., 2001). Thus, reduced FA and increased MD in the same brain tissue indicate the disrupted structure of myelin and axons along with abnormal levels of cellular fluid (Pfefferbaum and Sullivan, 2005). To conclude, the pattern of DTI metrics alternation in SCA3/MJD (decreased FA and increased MD, RD, and AD) reflects the dysfunction of the axonal function of white matter fibers and myelin degeneration rather than the process of primary demyelination. Neuropathological studies have observed neuronal loss and atrophy in the cerebellum, brainstem, and spinal cord (Riess et al., 2008) as well as the thalamus (Durr et al., 1996; Rüb et al., 2003; Tokumaru et al., 2003; Schols et al., 2015), basal ganglia (Durr et al., 1996; Yamada et al., 2008; Horimoto et al., 2011), and cerebral cortex (Paulson et al., 1997; Yamada et al., 2001; Seidel et al., 2012), which would eventually result in the loss of axons and the degeneration of neurons, providing a putative mechanism for the observed extensive WM involvement.

While it is true that DTI is very sensitive for the detection of microstructural degeneration, it is also important to note that DTI metrics such as FA or MD values can only provide comprehensive information about the integrity of brain WM (Concha, 2014). The sensitivity of DTI metrics to progressive brain changes in SCA3/MJD remains largely unexplored (Ashizawa et al., 2018). In addition, the commonly used DTI model might miss a subtle degeneration in WM and fail to distinguish crossing fibers (Ashizawa et al., 2018). These shortcomings can be compensated by more sophisticated diffusion models. Recently developed multicompartment techniques such as neurite orientation dispersion and density imaging model, by separating the signal deriving from different tissue compartments, have shown superior tissue and pathological specificity and might be particularly useful in detecting crossing fiber degeneration (Gatto et al., 2018; Broad et al., 2019). It is also worth paying attention to the continuous time random walk model, which could complement the traditional DTI model by providing supplemental information of brain tissue complexity (Ingo et al., 2014; Gatto et al., 2019). In addition, non-Gaussian diffusion models using fractal derivative model or fractional order model have been introduced to describe anomalous diffusion in human brain tissues with improved accuracy of MR imaging (Magin et al., 2008; Zhou et al., 2010; Liang et al., 2016).

Further animal studies and postmortem histology studies are still needed to validate the correlation of microstructural lesions and alteration of MRI metrics. Ideally, postmortem brain tissues are the closest ones for studying human diseases, whereas from the pre-clinical validation standpoint, animal models, especially mammalian models such as the classic ataxin-3-Q79 transgenic mouse that develops a progressive neurological phenotype of ataxia (Chou et al., 2008), the first humanized ataxin-3 knock-in mouse model, which combines the molecular and behavioral disease phenotypes with the genetic features of SCA3 (Switonski et al., 2015), and other knock-in mouse model that exhibits prominent aggregate pathology (Ramani et al., 2015), are all highly valuable for the clinical or the pre-clinical histopathologic validation of imaging data as well as the validation of disease mechanisms and treatment effect.

The morphological analysis of brain structure and DTI are mainly focused on the changes of brain anatomical structure at present. Although a direct and complete view of the relationship between brain structure and brain function is still largely unknown (Yang et al., 2016), the brain anatomical structure serves as the cornerstone that influences the brain function and disease progression, and the brain structural MRI researches in SCA3/MJD patients also promote the exploration of brain function.

## Blood Oxygen Level-Dependent Functional MRI

BOLD fMRI is a commonly used technique that can detect the spatiotemporal distribution of neural activity across the brain based on the detection of blood oxygenation level-dependent signal changes (Ogawa et al., 1990). In general, the differences in local brain activation patterns between patients with ataxia and normal controls were analyzed, with the presumption that the existence of differences may be related to neural circuit dysfunction in the patient group (Currie et al., 2013). There is a growing interest in exploring the utility of task-based and resting-state functional MRI in SCA patients for monitoring functional abnormalities in brain tissue.

Functional MRI patterns have detected a general dysfunction in the motor network in SCA3/MJD patients. Stefanescu et al. (2015) found that hand movement-related cerebellar activation was altered in SCA3/MJD patients, and significant activation in the ventral part of the dentate nucleus along with a reduced but non-significant reduction in the cerebellar cortex was detected when the subjects were instructed to open and close their right fist at a frequency of 1.66 Hz. Interestingly, in another fMRI study, cortical region activation was increased at 1 and 3 Hz but decreased at 5 Hz in 13 patients with early SCA3/MJD who were instructed to finger tap at 1, 3, and 5 Hz. Additionally, significant activation of subcortical regions, including the putamen, globus pallidus, and thalamus, was also discovered (Duarte et al., 2016).

The activation of cortical and subcortical regions in SCA3/MJD patients might represent a compensatory mechanism to overcome cerebellar dysfunction. Variations in BOLD fMRI signals depend on synaptic input (Lauritzen et al., 2012). Given that the ventral dentate nucleus is included in the pontocerebellar pathway (Glickstein and Doron, 2008), the increased activation of the ventral part of the dentate may suggest a possible compensatory recruitment of pontocerebellar areas to compensate for dysfunction in the spinocerebellar projections (Stefanescu et al., 2015), which is consistent with immunohistochemical data showing that afferent mossy fibers from pontine nuclei were preserved in SCA3/MJD patients (Koeppen et al., 2013). The decreases in the fMRI signal in SCA3/MJD patients may be due to decreased mossy fiber input to the cerebellum caused by the degeneration of the spinocerebellar tract. In addition, decreased activation at high frequency may hint that patients had surpassed the dynamic range of maintained function (Duarte et al., 2016). However, BOLD fMRI studies in patients with SCA3/MJD are very limited, and further studies are required to validate these hypotheses.

This inconsistency between the two studies may have been caused by differences in movement postures and frequency variation. In addition, discrepancies in the clinical characteristics of the patients studied, such as course of disease, CAG copy number, SARA scores, etc., may have also affected the results. Therefore, further prospective studies are needed to address the sensitivity and the specificity of these findings.

## Magnetic Resonance Spectroscopy

MRS is a non-invasive method that allows *in vivo* quantification of altered metabolite concentrations in brain tissue based on signals of hydrogen protons. MRS can provide biomarkers for neurological disorders even in cases where a lesion has not yet been observed in MR images (Duarte et al., 2012). The levels of NAA, Cho, mI, and Cr are the main metabolites that are focused on in most MRS studies. NAA concentration is an indicator of neuronal volume, viability, and integrity (Bachelard and Badar-Goffer, 1993; Tsai and Coyle, 1995), and the lack of NAA is associated with injury or loss of neurons and axons in various conditions (Bruhn et al., 1989; Kugel et al., 1992; Meyerhoff et al., 1994; Oppenheimer et al., 1995). Cho is the precursor of the neurotransmitter acetylcholine and membrane constituent phosphatidylcholine (Miller, 1991) and serves as an indicator of cell membrane and neurotransmitter metabolism (Lirng et al., 2012), mI is a marker of glial activation (Brand et al., 1993), which reflects neuronal injury and degeneration (Duarte et al., 2012), and Cr is an indicator of brain energy metabolism (Firbank et al., 2002; Lirng et al., 2012). The concentration of Cr is relatively stable under normal conditions; therefore, it often serves as a reference for comparisons (Lirng et al., 2012). Each metabolite shows a characteristic pattern at very specific resonance frequencies in MRS (Cousins, 1995). The neurometabolic spectrum detected in patients reflects the cell-specific changes in neurons or astrocytes, which cannot be evaluated by other neuroimaging methods.

### Neurochemical Alterations in Clinical SCA3/MJD Patients

Lower NAA and glutamate levels, reflecting the loss or dysfunction of neurons, were detected in the cerebellum and pons of SCA3/MJD patients (Adanyeguh et al., 2015), which was in agreement with the neuron loss found by neuropathological and structural imaging studies (Tokumaru et al., 2003; Lukas et al., 2006; Guimarães et al., 2013). The glial marker mI was elevated in the cerebellum and pons (Adanyeguh et al., 2015), reflecting the activation of glial cells in response to neuron degeneration (Brand et al., 1993; Allaman et al., 2011; Duarte et al., 2012). Total Cr (tCr) levels were also elevated in both the cerebellar vermis and pons in SCA3/MJD patients (Adanyeguh et al., 2015). The increase in tCr may be a compensatory response to maintain the function of the phosphocreatine/creatine kinase system to ensure the energy supply of brain tissue (Brewer and Wallimann, 2000). Moreover, the increased tCr may play a role in inhibiting the formation of free radicals and thereby strengthen neuroprotection (Adanyeguh et al., 2015). Similarly, increased tCr has also been detected in the brains of Huntington’s disease mouse models by 1H MRS (Zacharoff et al., 2012), suggesting that there may be similar abnormalities in brain energy metabolism in polyglutamine disease. Additionally, there is also evidence that the levels of glutamate and glutamine were reduced in cerebellar lesions, reflecting neuronal loss/dysfunction (Lopes et al., 2013; Adanyeguh et al., 2015).

In line with previous findings of decreased NAA/Cr ratios in the cerebellum (Lirng et al., 2012; Wang et al., 2012; Lopes et al., 2013; Chen et al., 2014; Huang et al., 2017; Peng et al., 2019) and deep WM (D’Abreu et al., 2009), a recent meta-analysis has also demonstrated a decrease in the NAA/Cr ratio in the cerebellum of SCA3/MJD patients compared to that of normal controls (Krahe et al., 2020). Reduced NAA/Cho, a marker reflecting brain metabolism (Meyerhoff et al., 1994; Ng et al., 1994; van der Grond et al., 1995), was also observed in the same areas (Lirng et al., 2012; Chen et al., 2014; Peng et al., 2019). Therefore, levels of NAA, suggestive of extensive neuronal and axonal dysfunction, might be regarded as an effective diagnostic marker of neurodegeneration in SCA3/MJD. The patterns with Cho/Cr have been more heterogeneous, with some studies demonstrating a reduced Cho/Cr ratio in the cerebellum (Peng et al., 2019; Krahe et al., 2020), but this was not obvious in other studies (Wang et al., 2012; Chen et al., 2014). Regarding these findings, further studies are needed to validate Cho as a metabolic marker (Krahe et al., 2020). The abnormal neurochemical ratios in different brain regions are summarized in Table 2.

**TABLE 2:** Abnormal neurochemical ratios in different brain regions.

Brain region	Abnormal neurochemical ratios	References
Cerebellum	NAA/Cr	Lei et al., 2011; Lirng et al., 2012; Wang et al., 2012; Lopes et al., 2013; Chen et al., 2014; Huang et al., 2017; Peng et al., 2019; Krahe et al., 2020
	Cho/Cr	Peng et al., 2019; Krahe et al., 2020
	NAA/mI	Joers et al., 2018
	NAA/Cho	Lei et al., 2011; Lirng et al., 2012; Chen et al., 2014; Peng et al., 2019
Brain stem	NAA/mI	Joers et al., 2018
Thalamus	NAA/Cr, NAA/Cho	Peng et al., 2019
Deep white matter	NAA/Cr, Cho/Cr	D’Abreu et al., 2009

### Neurochemical Profile Abnormalities Precede Clinical Manifestations

A recent profound study detected reduced NAA/mI ratios in the pons and cerebellum in SCA3/MJD patients whose estimated disease onset was within 10 years (Joers et al., 2018). Significantly reduced NAA/Cr and NAA/Cho ratios in the cerebellar hemispheres and vermis were also observed in the early stages of SCA3/MJD (SARA score < 10) (Lirng et al., 2012). Using 18F-fluorodeoxyglucose positron emission tomography, Soong and Liu detected abnormal FDG consumption levels in the cerebellum, brainstem, and cerebral cortex in asymptomatic carriers of SCA3/MJD (Soong and Liu, 1998). Additionally, in other SCA diseases, such as SCA1, SCA2, and SCA6, neurochemical changes were also found to precede the onset of clinical manifestations (Guerrini et al., 2004; Wang et al., 2012). Potentially, MRS can reveal early metabolic/cellular changes in various SCAs, which are likely to occur before cerebellar signs are obvious, even before brain atrophy, which indicates that the measurement of neurochemical changes in brain tissue by MRS may be superior to clinical observation in particular circumstances. The discovery of early neurochemical changes in non-ataxia mutant gene carriers could not only help to illustrate the underlying pathophysiology of SCAs but also provide a window for early intervention preceding irreversible brain damage. However, neurochemical abnormalities in the premanifest stage of SCAs remain largely unexplored (Joers et al., 2018). More studies at the preclinical stage in patients with different SCA genotypes are needed to further support the potential of neurochemical metabolites as prognostic biomarkers.

### Neurochemical Metabolites Are Associated With Clinical Features

Similar to morphometric neuroimaging, neurochemical alterations, including NAA/Cr, Cho/Cr, or NAA/Cho ratios, correlated with disease duration, SARA scores (Lirng et al., 2012; Wang et al., 2012; Huang et al., 2017), ICARS scores (Peng et al., 2019), and genetic data (Wang et al., 2012; Peng et al., 2019) in SCA3/MJD patients, suggesting that MRS can serve as a biomarker to monitor the progression of ataxia and evaluate the response to therapeutic interventions, even in the early stages of SCA3. Importantly, an animal study of SCA1 has validated the sensitivity and the specificity of MRS as a non-invasive technique to reflect the extent of recovery from neurodegeneration even better than standard motor behavioral assessment (Öz et al., 2015). Moreover, the test–retest reproducibility of neurochemical profiles by MRS was demonstrated to be reliable not only within site (Terpstra et al., 2016) but also between sites (Deelchand et al., 2015; van de Bank et al., 2015), which is crucial for its utility in future multisite trials. However, there have also been contrasting findings that make these inferences inconclusive; these studies showed that CAG repeat length and clinical features (such as SARA scores and course of disease) were not related to the concentrations of any neurochemical metabolites (D’Abreu et al., 2009; Adanyeguh et al., 2015; Joers et al., 2018). The detailed correlations of neurochemical ratio abnormalities and clinical data are summarized in Table 3. A recent study using a novel multi-variate approach, distance-weighted discrimination, allows a combination of multiregional neurochemical profiles to estimate time to disease onset, and the correlation between the DWD-based MRS scores and SARA and duration in SCA3 patients has suggested a regional effect of neurochemical profiles (Joers et al., 2018). Further studies would be of interest to determine whether neurochemical metabolite levels are a useful biomarker of disease progression, and the relationship between regional neurochemical levels and brain pathology deserves more attention.

**TABLE 3:** The correlation of neurochemical ratio abnormalities and clinical data.

Clinical data	Correlation	Cerebellum			Deep white matter	
		NAA/Cr	Cho/Cr	NAA/Cho	NAA/Cr	Cho/Cr
DD	Relevant	Huang et al., 2017; Peng et al., 2019	Peng et al., 2019	Lirng et al., 2012	/	/
	Irrelevant	Lirng et al., 2012	Lirng et al., 2012	Peng et al., 2019	D’Abreu et al., 2009	D’Abreu et al., 2009
SARA	Relevant	Lirng et al., 2012; Wang et al., 2012	Lirng et al., 2012	Lirng et al., 2012	/	/
	Irrelevant	/	/	/	/	/
ICARS	Relevant	Peng et al., 2019	Peng et al., 2019	/	/	/
	Irrelevant	/	/	Peng et al., 2019	D’Abreu et al., 2009	D’Abreu et al., 2009
CAG	Relevant	Peng et al., 2019	/	Peng et al., 2019		
	Irrelevant	Wang et al., 2012; Huang et al., 2017	/	/	D’Abreu et al., 2009	D’Abreu et al., 2009

*DD, disease duration.*

## Conclusion and Perspectives

In conclusion, this review summarized the value of MR imaging techniques in detecting structural and functional changes in patients with SCA3/MJD and demonstrated the feasibility of detecting neuroimaging abnormalities in the preclinical stage of this disease, as well as their correlations with clinical features and disease severity. These MR imaging abnormalities identified in clinical and preclinical SCA3/MJD patients provide significant insights into the pathogenesis of the disease. Morphometric MR imaging has been proven to be an effective method of monitoring brain atrophy. DTI studies provide comprehensive information about the integrity of brain WM, and metabolites related to neuronal loss, glial cell activation, and abnormal brain energy metabolism have been detected by MRS. Both DTI and MRS abnormalities indicate that the synaptic function or density might have been disrupted, while the sensitivity of DTI metrics, MRS, and functional MR imaging alterations to progressive changes of brain tissue in SCA3/MJD remains unclear. In addition, due to the heterogeneity in imaging parameters across different studies, such as the magnetic flux density, sequences of image acquisition, and operation protocols, the accuracy of a direct comparison between various research results is relatively limited. Histopathologic and animal studies are further needed to validate the correlation between MR imaging and pathological findings. A combination of multiple data sets from magnetic resonance imaging and clinical scales or even body fluid biomarker might perform better in the identification of potential biomarkers in SCAs. Validated neuroimaging biomarkers are of great value in guiding patient eligibility, patient stratification, and tracking therapeutic response in clinical trials. Standardized and systematic analyses of demographics and clinical and genetic data could contribute to defining the sensitivity of neuroimaging biomarkers. In light of this fact, multisite longitudinal prospective studies utilizing multimodal MR imaging techniques in a large cohort of patients as well as preclinical mutation carriers with SCA3/MJD are crucially needed to validate the capability of MR imaging in the diagnosis and the differentiation of SCA subtypes, predicting the age of disease onset in preclinical individuals, monitoring disease progression even before the onset of symptoms, and assessing the therapeutic effect of clinical treatments.

## Author Contributions

NW wrote the manuscript. ZC, LW, and BT helped revise the manuscript. HJ agreed to be accountable for the content of the work. All the authors approved the final version of the manuscript.

## Conflict of Interest

The authors declare that the research was conducted in the absence of any commercial or financial relationships that could be construed as a potential conflict of interest.
